# Body image perceptions and symptoms of disturbed eating behavior among children and adolescents in Germany

**DOI:** 10.1186/s13034-018-0216-5

**Published:** 2018-01-24

**Authors:** Kathrin Schuck, Simone Munsch, Silvia Schneider

**Affiliations:** 1 Mental Health Research and Treatment Center, Ruhr-University Bochum, Massenbergstrasse 9-13, 44787 Bochum, Germany; 2 Department of Psychology, Clinical Psychology and Psychotherapy, University of Fribourg, Rue P.A. de Faucigny 2, 1700 Fribourg, Switzerland

**Keywords:** Body image, Eating disorders, Cognitive distortion, Children, Adolescents

## Abstract

**Theoretical background:**

Body image distortions such as perception biases are assumed to be precursors of eating disorders (ED). This study aims to investigate body image perceptions and symptoms of disturbed eating behavior among a sample of 11–17 year-old students in Germany.

**Methods:**

A cross-sectional survey study was carried out among 1524 students of twelve secondary schools from all school types in North Rhine-Westphalia (Germany). A naturalistic photograph-rating consisting of photographs of young women’s bodies was used to examine children’s perceptions of female bodies (i.e., perceived average body size and perceived ideal body size of young women). Also, symptoms of disturbed eating behavior were examined.

**Results:**

Compared to statistical data, children and adolescents underestimated the average body size of young women by more than two BMI-points (estimated average BMI = 20), with no differences between boys and girls. Also, girls and boys generally held a slim female thin-ideal (perceived ideal BMI = 19.5), which is nearly three BMI-points below the average body size in the young female population. Girls showed a slightly stronger female thin-ideal than boys. Among all subgroups, early-adolescent girls (13–14 years) displayed the strongest thin-ideal internalization. Nearly one-third of this group perceived a BMI below 18 as ideal female body size. Symptoms of disturbed eating behavior were common among youth and most frequent among adolescent girls (15–17 years). Girls who displayed a bias towards underestimation of female body size and girls who displayed an underweight female thin-ideal were more likely to report harmful dieting behaviors and psychological distress associated with eating, body, and weight.

**Conclusions:**

This study found that 11–17 year-old girls and boys do not show accurate judgements regarding the average body size of young women. Instead, there is systematic and significant underestimation, indicating considerable perception biases, which may constitute a risk factor for the development and maintenance of ED. Symptoms of disturbed eating behavior were common, especially among girls, and associated with body-related perceptions. Future research will need to clarify the severity and course of these symptoms.

## Background

Body image is a multi-dimensional concept, which describes how we think, feel, perceive, and act with regard to our bodies. Adolescence constitutes a critical period for the development of a healthy or unhealthy body image [1]. A large number of studies have consistently shown that a negative body image, typically measured as body dissatisfaction, is associated with disturbed eating patterns among adolescents [2–6] and one of the strongest risk factors for the development of eating disorders (ED) [7, 8] and other adverse psychological outcomes such as depression [9–11].

Body image disturbances are key characteristics of eating disorders (EDs) such as anorexia nervosa and bulimia nervosa and encompass distortions in cognition, affect, perception, or behavior related to body weight or shape [12]. They may refer to negative thoughts or negative evaluation regarding one’s own body, negative affect in response to one’s own body, misperception of body-related stimuli, and specific body-related behaviors (e.g., checking or avoidance). In Western societies, body image disturbances including body dissatisfaction are pervasive problems. Particularly among women, the desire for thinness is so prevalent that it is considered a normative discontent [13]. A growing body of evidence suggests that this body-related discontent may apply to a similar extent to children and adolescents. A large number of studies has shown that body image disturbances (e.g., body dissatisfaction, discrepancy between one’s actual and one’s ideal body size, weight and shape concerns) frequently occur even before puberty and are reported by up to 50% of children and adolescents [5, 7, 14–23].

Similarly, a growing body of research suggests that symptoms of disturbed eating behavior are common among youth. In a large German study among 7498 students (11–17 years old), nearly one quarter (21.9%) showed symptoms of EDs (e.g., concerns about loss of control over eating, self-induced vomiting, rapid weight loss in the last 3 months). Girls were significantly more often affected than boys (28.9% vs. 15.2%) [24]. Similarly, a study conducted in the United States among 1739 female students (12-18 years) reported that disordered eating attitudes and behaviors (e.g., dieting, binge eating) were present in 27% [25]. Similar numbers have been reported by other studies [17, 20, 21, 26, 27]. The outcomes of eating-disordered attitudes and behaviors in adolescence are severe. Prospective studies show that body dissatisfaction and early ED symptoms (e.g., body image distortions, weight concerns) predict eating-disordered behavior, onset of ED, depressive symptoms, overweight, and obesity in adulthood [3, 26, 28, 29].

While there is consistent evidence that body image disturbances in terms of dysfunctional cognitions (e.g., body dissatisfaction), negative affect (distress in response to weight or shape), and behavioural measures (e.g., symptoms of disturbed eating behavior) already appear in children and adolescents, few data is available regarding body image perceptions. Recent studies have used pictorial figure rating scales to examine body image perceptions, which typically consist of a series of abstract figures ranging from underweight to overweight (for an overview, see [30]). Up to this point, only a handful of studies have employed figure rating scales displaying naturalistic human bodies [31–35] and only one of these studies has been conducted among children [35]. While this study reported discrepancies between children’s own body image and ideal body image, normative perceptions of human body sizes (e.g., the ability to correctly perceive human bodies in terms of normality) have not been investigated.

Perceptual distortions may play an important role in the development of EDs [8, 36]. Perceptual distortions are considered a type of cognitive bias, which describe systematic errors in the processing of information (i.e., information processing biases). There is accumulating evidence that cognitive biases may influence the onset and maintenance of eating-related pathology in adolescence and early adulthood [37–42]. Cognitive biases may occur in different domains such as attention, perception, or memory and may foster symptoms of mental disorders, because they determine what people notice, attend to, and remember. In ED, perceptual biases related to body weight or shape (e.g., systematic misperceptions or judgement errors) have been proposed to reinforce disturbed body image experiences [43]. For example, underestimating the average body size may result in a larger perceived discrepancy between oneself and the norm, thereby increasing body dissatisfaction and weight and shape concerns.

The present study aimed to examine normative perceptions (perceived average body size) and thin-ideal perceptions (perceived ideal body size) of female bodies^1^ among 11–17 year old children and adolescents using a naturalistic photographic figure rating. Furthermore, symptoms of disturbed eating behavior were studied in relation to these perceptions. We hypothesized that children and adolescents would systematically underestimate the average female body size in comparison to the average statistical body size. We also expected that children and adolescents would display a slim female thin-ideal. In addition, we expected that symptoms of disturbed eating behavior would be associated with a bias towards underestimation of female body size and an underweight thin-ideal.

## Methods

### Participants and procedure

Study participants were 1524 children and adolescents aged between 11 and 17 years who were recruited from 12 secondary schools from all school types in North Rhine-Westphalia, Germany. Schools were selected from a larger pool of schools and school principals were contacted by telephone by research assistants, who informed them about the study. A total of 119 schools were initially contacted and 12 schools agreed to distribute short questionnaires during school hours to all students in German grades 5–10 (US grades 6–11). Parents received written information about the school’s participation in the study as well as information about the procedure and aim of the study. All parents were informed that participation in the study was voluntary and received a form to withdraw their child from study participation and a return envelope (‘passive consent’). Five children were excluded by their parents from study participation. Data collection took place between April and July 2015. Before the assessment, children were informed about the aim of the study and that participation was voluntary. They received information about the general topic (eating behavior and body image) and the procedure. Questionnaires were filled in anonymously in the presence of an instructed teacher and a research assistant. The study was approved by the ethics committee of the Faculty of Psychology of the Ruhr-University Bochum, Germany.

### Measures

#### Photograph-rating of female bodies

To measure body size perception of female bodies, a photograph figure rating based on the Stunkard Figure Rating Scale [44] was used. The original rating scale consists of silhouette drawings of female bodies ranging from very thin to very large. In the present study, a photographic figure rating was developed using body photographs of women’s bodies. As human bodies are quite diverse, the rating consisted of a total of 24 photographs of women varying in body mass index (BMI). The photographs depicted female university students from neck down in different standardized perspectives wearing standardized, beige underwear in front of a white background. The pictures were taken at the Ruhr-University Bochum for the purpose of another study on body image conducted by the first author (material is available upon request). All photographs were released by the former study participants through written consent to be used for research purposes.

A systematic review on pictorial figure rating scale [30] noted that scales often depict unrealistic representations of human body forms (e.g., contour drawings or computerized figures with disproportionately sized or poorly defined body features). Hence, more naturalistic representations of human bodies are needed to increase ecological validity in the assessment of body images. An additional potential limitation of previously used photographic figure rating scales is that few response choices are provided. In previously used scales, one individual body represents one body size, which may be confounded with other variables such as perceived attractiveness, hip-to-waist ratio, or proportions between body features. This methodological artifact (“scale coarseness”) limits measurement precision and increases the likelihood of measurement errors [30]. The present study aimed to overcome these methodological limitations by using a photographic figure rating, which consisted of several sets of naturalistic photographs of young women’s bodies (four sets each displaying six bodies with varying BMIs), resulting in multiple response choices.

To assess body image perceptions among youth, children and adolescents were presented with four photographic figure rating scales, each consisting of six female bodies differing in BMI from underweight to overweight. Each scale depicted six bodies with the following BMIs: 1) BMI between 16.5 and 18 (underweight), 2) BMI between 18.5 and 20, 3), BMI between 20 and 21 4) BMI between 21.5 and 23, 5) BMI between 23 and 25, and 6) BMI between 25 and 28 (overweight). BMIs were presented in ascending and descending order (the order was counterbalanced within the photograph-rating). The four sets depicted bodies from different perspectives (i.e., the first scale depicted bodies from front view, the second from back view, the third from 90-degree side view, and the fourth from 45-degree side view).

Children and adolescents were asked the following: “Please indicate which of these body sizes is most similar to the ideal body of a young woman”, and “Please indicate which of these body sizes is most similar to the average body of a young woman”. A mean score and a corresponding BMI for the two variables *average body size* and *ideal body size* was calculated based on the scores endorsed on the four photographic rating scales. To examine perception biases, the perceived average body size of young women reported by children and adolescents was compared to data of the average body size of 18–25 year old women in Germany reported by the Federal Statistical Office. Moreover, we calculated the percentage of children who correctly estimated the average body size of young females, defined as frequently selecting category 4 (BMI: 21.5–23), which displays body sizes closest to the statistical average body size of young females (i.e., selecting category 4 on at least three out of four times on the photographic rating scales). Correspondingly, we also calculated the percentage of children who displayed a bias towards underestimation (i.e., selecting lower BMI categories on average) and a bias towards overestimation (i.e., selecting higher BMI categories on average). To examine pervasive thin-ideal perceptions, we calculated the percentage of children who displayed an underweight thin-ideal, defined as frequently selecting category 1 (BMI: 16.5–18), which displays underweight body sizes according to the World Health Organization (i.e., selecting category 1 at least three out of four time on the photograph rating scales).

To examine construct validity of the photograph-rating, we conducted an “expert-rating” among ten mental health professionals (5 female 5 male). Herefore, a convience sample of ten licensed psychotherapists working at the Mental Health Research and Treatment Center of Ruhr-University Bochum was asked to examine the photographic material used in the present study. Mean age of psychotherapists was 32.1 years (SD = 3.9). All psychotherapists had experience in treating eating disorders, but none of them considered himself to be an expert in this area. The aim was to present a proof-of-concept and an indication of face-validity by examining whether mental health professionals would be able to correctly order the female body photographs by increasing BMI and if they would be able to correctly perceive under- and overweight. Each psychotherapist was presented with the four rating scales consisting of six female bodies each. For the present purpose, the female bodies were presented in quasi-random order. Psychotherapist were asked to re-order the photographs per scale by increasing body weight. Also, they were asked to indicate whether they perceived any of the bodies to be under- or overweight. To examine construct validity, we calculated Cohen’s kappa to compare agreement between the correct ranking order and the psychotherapist’s ranking order [cf. 31]. In addition, we conducted sensitivity and specificity analyses. Kappa coefficients ranged between .65 and .90 with an average of .79, which indicates good to excellent agreement between actual body size and the psychotherapist’s perception of body size. Sensitivity and specificity scores were generally high, indicating that psychotherapists were correctly able to perceive under- and overweight. With the exception of one psychotherapist who never recognized underweight, sensitivity scores for underweight ranged between 75 and 100% (on average 85%), indicating that underweight was correctly perceived in the majority of cases. Specificity scores for underweight were 100% among all psychotherapists, indicating that non-underweight was never falsely perceived as underweight. Sensitivity scores for overweight were 100% for all experts, indicating that overweight was always correctly perceived as overweight. Specificity scores for overweight ranged between 85 and 100% (on average 97%), indicating that non-overweight cases were rarely perceived as overweight. In sum, the present expert-rating indicates good construct validity. In addition, previous research has shown good test–retest validity of photographic figure rating scales as well as good convergent validity with other established measures of eating disorders [31–34].

#### Eating-related behaviors

To assess eating-related behaviors, participants were asked to respond to the following items previously applied in a large survey study by Micali and colleagues [cf. 20]: “In the past 3 months, did you do any of the following things to influence your weight: “eating less during meals”, “skipping meals”, “fasting (e.g., not eating for the entire day or almost the entire day)”, “exercising to loose weight or to prevent weight gain”, “self-induced vomiting”, and “taking diet pills or laxatives”. To assess symptoms of disturbed eating behavior, participants were asked to respond to the following items [cf. 20]: “Do you feel fat, even though other people tell you that you are not?”, “Are you terrified of gaining weight or getting fat?”, “Do you avoid certain types of food because you fear weight gain?”, “Do you feel upset about your weight or shape?”, “Do you feel distressed after eating too much?”, “Do you have episodes of binge eating, in which you eat a very large amount of food?”, “Do you ever loose control over eating?” Response options for all items were no (0) or yes (1). The items are based on DSM-IV and ICD-10 criteria for ED and they are likely to reflect broader early ED phenotypes, indexing risk for clinical disorders [20]. Previous research has shown that these ED symptoms are associated with psychological outcomes such as social impairment, family burden, and emotional and behavioral disorders. The items have been selected, as they have demonstrated concurrent and predictive validity [20] and can be more easily administered to children than other measures of ED pathology, which may require more complex answers.

#### Scales indexing risk for ED

In addition to the aforementioned items, we included two scales indexing risk for ED with well-established psychometric properties. The subscale shape concern of the Eating Disorder Examination Questionnaire (EDE-Q; Fairburn & Beglin [45]) consists of eight questions measuring body dissatisfaction and shape concerns. The items refer to the past 28 days and are rated using seven point forced-choice format (1–7). The EDE-Q has a high internal consistency (α = .97) and good convergent validity [46]. Responses were added into a mean score with higher score reflecting higher levels of body dissatisfaction and shape concerns. The Sociocultural Attitudes towards Appearance Scale (SATAQ-G, Knauss et al. [47]) assesses the recognition and endorsement of societal appearance standards. The questionnaire consists of 16 items and three subscales: internalization, perceived pressure and awareness of sociocultural appearance standards. Response options range from 1 (strongly disagree) to 5 (strongly agree). Reliability of the subscales is high (Cronbach’s alphas = .92–.96; Thompson et al. [48]). The questionnaire has acceptable concurrent validity [47]. Responses were added into a mean score with higher scores reflecting a stronger recognition and endorsement of societal appearance standards.

#### Strategy for analyses

Analyses were conducted for the total sample and separately for girls and boys. To distinguish between developmental stages, participants were divided into three age groups (pre-adolescents: 11–12 years, early-adolescents: 13–14 years; adolescents: 15–17 years) [cf. 49]. Descriptive statistics (means, standard deviations, frequencies) were used to examine sociodemographic characteristics as well as variables of interest. Statistical comparisons between groups were based on independent sample t-tests for continuous variables and Chi square tests for categorical variables. To examine associations between body image perceptions (average body size and ideal body size) and symptoms of disturbed eating behavior, we compared the frequency distributions of symptoms between samples using Chi square tests. First, we compared girls who displayed a strong bias towards underestimation of average female body size to girls without a strong bias towards underestimation. Therefore, the sample was split in tertiles based on the perceived average female body size on the photograph-rating. Girls scoring within the lowest tertile were compared to girls scoring within the highest tertile.^2^ Second, we compared girls who displayed an underweight thin-ideal to girls who did not display an underweight thin-ideal (defined as frequently selecting a BMI below 18 as ideal body size vs. other responses on the photograph-rating).

## Results

### Descriptive analyses

Table 1 displays descriptive statistics for the total group and for girls and boys separately. On average, participants were 13.6 years (*SD* = 1.8). A total of 828 (55.1%) were girls and 676 (44.9%) were boys. All participants were divided into three developmental groups, respectively pre-adolescents (n = 482, 32%), early-adolescents (n = 495, 32.9%), and adolescents (n = 527, 35.0%). A total of 597 children and adolescents (39.9%) attended the highest (*Gymnasium*) of three German school forms, 151 (10.1%) attended the lowest school form (*Hauptschule*). In comparison to data from the Federal Statistical Office in Germany [50], the characteristics of the present sample resemble population characteristics of students in North-Rhine Westphalia (Germany’s most populous state), in terms of school type and age. However, boys were somewhat underrepresented (44.9% vs. 51.0%) in the present study.

**Table 1:** Descriptive statistics of children and adolescents in the present study

	Total sample	Girls	Boys
Gender (n, %)			
Female	828 (55.1)		
Male	676 (44.9)		
Age group (n, %)			
Pre-adolescent (11–12 years)	482 (32.0)	248 (30.2)	231 (34.5)
Early-adolescent (13–14 years)	495 (32.9)	277 (33.7)	214 (32.0)
Adolescent (15–17 years)	527 (35.0)	297 (36.1)	224 (32.5)
School form (n, %)			
High	597 (39.9)	383 (46.9)	209 (31.2)
Medium	750 (50.2)	377 (46.1)	367 (54.9)
Low	151 (10.1)	57 (7.0)	93 (13.9)
Age (M, SD)			
	13.6 (1.8)	13.7 (1.7)	13.6 (1.8)

*M* mean, *SD* standard deviation

### Body image perceptions

With regard to the average body size, children and adolescents endorsed a mean score of 2.7 on the photographic figure rating scale, which corresponds to a BMI of approximately 20.0. There was no statistically significant difference between girls and boys (M_girls_ = 2.7, M_boys_ = 2.7, t = − 1.8, p = .07, *d* = .09). According to the Federal Statistical Office, the average BMI of a young woman (20–25 years) in Germany was 22.4 in the year 2013 [51]. This comparison shows that children and adolescents underestimate the average body size in the population by more than two BMI-points. Only 8.1% of children correctly estimated the average female body size (defined as frequently selecting category 4 on the photograph-rating, which depicts BMIs between 21.5 and 23). In contrast, 88.1% showed a bias towards underestimation of the average female body size, while only 3.8% showed a bias towards overestimation.

With regard to ideal body size, children and adolescents perceived the ideal body size of a young woman to be 2.1 on the photographic figure rating scale, which corresponds to a BMI of approximately 19.5. Comparison between perceived body size ideal and actual body size according to statistical data showed that children and adolescents hold a slim female thin-ideal, which deceeds the average body size in the population by nearly three BMI-points. There was a slight, but statistically significant difference between girls and boys (M_girls_ = 2.0, M_boys_ = 2.2, t = − 5.1, p < .001, *d* = .27), indicating that the perceived ideal body sizes for young females was slightly lower among girls than among boys.

In addition, we examined the percentage of children and adolescents who display an underweight thin-ideal (i.e., frequently endorsing a BMI below 18 as ideal body size on photograph-rating). Table 2 displays percentages by gender and age group. The proportion of children and adolescents who hold an underweight thin-ideal was generally higher among girls compared to boys (24.9% vs. 16.8%). Among all subgroups, early-adolescent girls held the strongest thin-ideals. Nearly one-third (30.1%) of 13–14 year old girls perceived a BMI below 18 (underweight) as ideal body size. Among 15–17 year old girls, a quarter (24.7%) perceived a BMI below 18 (underweight) as ideal body size.

**Table 2:** Percentage of children and adolescents who display an underweight thin-ideal by gender and age group

	Total sample	Girls	Boys
Pre-adolescents (11–12 years)	19.2	18.6	20.0
Early-adolescents (13–14 years)	24.7	30.1	17.4
Adolescents (15–17 years)	19.6	24.7	12.6
Total sample (11–17 years)	21.5	24.9	16.8

### Symptoms of disturbed eating behavior

Table 3 displays the frequency of symptoms of disturbed eating behavior for the total group as well as by gender and age group. Symptoms of disturbed eating behavior were common and generally higher among girls. Feeling fat, feeling upset about weight or shape, restrictive eating, exercising for weight control, and distress after eating were reported by a quarter to a third of all children and adolescents. In addition, unhealthy eating behaviors such as skipping meals or fasting were reported by a substantial proportion of youth (21.8 and 15.3%, respectively), especially among adolescent girls (37.4 and 27.7%, respectively). Episodes of binge eating and loss of control over eating were also reported quite frequently by youth (16 and 11%, respectively), again, especially by adolescent girls (24.9 and 14.2%, respectively). Harmful compensatory behaviors (self-induced vomiting or taking dieting pills or laxatives) were generally rare among youth, although a significant percentage of early-adolescent and adolescent girls reported self-induced vomiting within the last 3 months (4.8 and 4.1%, respectively).

**Table 3:** Symptoms of disturbed eating behavior among children and adolescents by gender and age group

Item	Total sample (%)	Girls			Boys		
		11–12 (years) (%)	13–14 (years) (%)	15–17 (years) (%)	11–12 (years) (%)	13–14 (years) (%)	15–17 (years) (%)
Eating less during meals	34.8	35.1	42.0	46.7	30.0	26.1	22.7
Skipping meals	21.8	13.9	26.8	37.4	14.2	20.5	13.0
Fasting	15.3	11.3	16.6	27.7	9.0	14.2	8.3
Exercising for weight control	58.0	58.7	66.8	65.1	51.6	50.9	62.3
Self-induced vomiting	2.8	1.3	4.8	4.1	2.3	2.4	.9
Taking diet pills or laxatives	1.7	.4	1.5	2.8	1.4	1.4	2.3
Feeling fat	36.0	44.8	48.2	53.6	22.6	21.4	14.5
Terrified of gaining weight	21.9	26.8	25.1	30.7	18.2	13.9	11.9
Avoidance of certain food	20.6	17.9	19.9	28.2	20.7	20.2	15.1
Feeling upset about weight or shape	36.1	38.3	41.9	51.6	24.8	27.3	24.1
Distress after eating	25.7	27.2	32.0	38.2	16.8	18.0	15.3
Episodes of binge eating	16.0	14.3	19.0	24.9	9.1	9.5	14.3
Loss of control over eating	11.0	13.2	14.2	14.2	5.5	6.6	10.1

Results are displayed as absolute percentages

### Associations between body image perceptions and symptoms of disturbed eating behavior

Associations between perceived average body size and perceived ideal body size and symptoms with disturbed eating behavior among girls are displayed in Table 4. Girls who displayed a strong bias towards underestimation were more likely to report skipping meals (29.7% vs. 23.2%), being terrified of gaining weight (32.8% vs. 22.8%), avoidance of certain food (26.7% vs. 19.3%), feeling upset about weight or shape (48.9% vs 39.3%), distress after eating (38.1% vs. 27.2%, respectively), and perceived loss of control over eating (16.9% vs. 10.7%) compared to girls who did not display a strong bias towards underestimation of female body size. In line with this, they also displayed higher levels of shape concerns (16.4 vs. 13.3, *p* < .01) and a stronger endorsement of societal appearance standards (44.0 vs. 41.5, *p* < .05) compared to girls did not display a strong bias towards underestimation of female body size.

**Table 4:** Frequency distributions of symptoms among girls in relation to perception biases and an underweight thin-ideal

Symptom	Girls with strong bias towards underestimation (%)	Girls without strong bias towards underestimation (%)	p value	Girls with underweight thin-ideal (%)	Girls without underweight thin-ideal (%)	p value
Eating less during meals	42.7	37.3	.11	44.5	40.5	.19
Skipping meals	29.7	23.2	.05	32.4	25.4	.04
Fasting	21.4	17.6	.14	22.1	18.0	.13
Exercising for weight control	62.5	62.7	.51	65.6	62.0	.21
Self-induced vomiting	4.2	3.2	.36	6.9	2.7	.01
Taking diet pills or laxatives	.7	1.6	.25	1.6	1.6	.61
Feeling fat	47.9	45.5	.31	52.9	47.5	.12
Terrified of gaining weight	32.8	22.8	< .01	31.0	26.3	.13
Avoidance of certain food	26.7	19.3	.02	29.3	20.3	<.01
Feeling upset about weight or shape	48.9	39.3	.01	50.0	42.1	.04
Distress after eating	38.1	27.5	< .01	41.7	30.2	<.01
Episodes of binge eating	17.5	21.1	.15	17.5	20.6	.20
Loss of control over eating	16.9	10.7	.02	14.2	13.2	.40

p values pertain to Chi square tests

Girls who displayed an underweight female thin-ideal were more likely to report skipping meals (32.4% vs. 25.4%), self-induced vomiting (6.9% vs. 2.7%), avoidance of certain food (29.3% vs. 20.3%), feeling upset about weight or shape (50.0% vs. 42.1%), and distress after eating (41.7% vs. 30.2%) compared to girls who did not display an underweight female thin-ideal. In line with this, they also displayed higher levels of shape concerns (16.8 vs. 14.1, *p* = .02) and a stronger endorsement of societal appearance standards (47.2 vs. 41.8, *p* < .001) compared to girls who did not display an underweight thin-ideal.

## Discussion

The present study aimed to answer the question how accurate children and adolescents judge body sizes of young females in terms of normality and if there is a general bias towards underestimation of female body size among youth. Using a photograph-rating consisting of sets of naturalistic photographs of young women’s bodies, body image perceptions (i.e., perceived average female body size and perceived ideal female body size) were examined in a large sample of 11–17 year old German students.

The present study is the first to show that children and adolescents considerably underestimate the average female body size when judging naturalistic photographs of young female bodies. On average, they underestimated the average body size of a young woman by more than two BMI-points (i.e., they perceived the average BMI of a young woman to be approximately 20, while the average BMI of the reference population is 22.4). Perceptual biases such as normative misperceptions have been found to play an important role in several health-related behaviors such as uptake of smoking or drinking among youth [52, 53]. Similarly, perceptual body-related distortions may influence eating-related attitudes and behaviors by increasing the perceived discrepancy between oneself and the norm, resulting in body dissatisfaction and weight and shape concerns. Research supports these assertions by showing that women who felt discrepant from the norm show more symptoms of ED [54], which may results in more extreme and maladaptive dieting behaviors to achieve an unrealistic and often unattainable body size.

Furthermore, the present study showed that girls and boys generally held a slim female thin-ideal (i.e., they perceived the ideal BMI of a young woman to be approximately 19.5), which represents the lowest quartile of a healthy BMI range (18.5–25). Yet, a substantial proportion of children and adolescents displayed an underweight thin-ideal (24.9% among girls, 16.8% among boys). The results are in line with previous studies. Connolly, Slaughter, and Mealey [55] showed that already 6-year olds have a systematic preference for underweight body shapes. Similarly, Brown and Slaughter [15] showed that children and adolescents across all age groups rate thin female bodies as more attractive than normal bodies. Schneider and colleagues [21] showed that adolescent girls desired a body shape for themselves corresponding to underweight. Similar strong thin-ideals have been observed in adult women [56–59]. In sum, a large body of research indicates that the sociocultural thin-ideal is internalized by a large proportion of the Western population including children and adolescents. The results of the present study strengthen and extend findings of previous studies using pictorial instead of photographic figure rating scales, which may be limited by methodological shortcomings.

Finally, the present study showed that symptoms of disturbed eating behavior among youth were quite common, especially among female adolescents. Feeling fat, feeling upset about weight or shape, restrictive eating, exercising for weight control, and distress after eating were reported by a quarter to a third of all children and adolescents. Also, a substantial proportion of youth reported unhealthy eating behaviors such as skipping meals or fasting (21.8 and 15.3%, respectively), episodes of binge eating (16%), and perceived loss of control over eating (11%). The results are in line with previous research showing that symptoms of disturbed eating behavior are common among youth [17, 20, 21, 24–27]. Importantly, body image perceptions were associated with disordered eating behaviors among youth. Girls who displayed a strong bias towards underestimation of the average female body size and girls who displayed an underweight thin-ideal were more likely to report harmful dieting behavior (e.g., skipping meals, self-induced vomiting) and psychological distress associated with eating and own body weight (e.g., being terrified of gaining weight, feeling upset about weight or shape, distress after eating). Also, they showed significantly elevated scores on well-established measures indexing risk for ED (i.e., higher levels of shape concerns and a stronger recognition and endorsement of societal appearance standards). These associations indicate that both perceptional biases as well as the internalization of a pervasive thin-ideal may constitute risk factors for the onset and maintenance of ED among youth.

In addition, differences between boys and girls were examined. It is reasonable that both boys and girls hold body images, not only for their own but also for the opposite sex (i.e., ideas about how males and females should look like). With regard to the perceived average female body size, boys and girls did not differ (both underestimated the average female body size to a similar extent). However, with regard to the perceived ideal female body size, girls showed a slightly lower thin-ideal than boys. Previous studies found similar results among adults, showing that men and women differ in attractiveness ratings of female body size, with males being less stringent about female body size than females [60–62]. However, it should be noted that the present study only examined the female body ideal (i.e., thin-ideal), while the male body ideal (i.e., muscular ideal) has not been examined. Therefore, it remains unclear whether females in general are more susceptible than males to adopt and internalize sociocultural body ideals or whether females and males internalize gender-specific sociocultural body ideals to a similar extent. For a comprehensive picture, body ideals of both male and female bodies should be compared between boys and girls.

In addition, differences between developmental groups were examined. Interestingly, the group of early-adolescent girls most often displayed an underweight thin-ideal. Nearly one-third of 13–14 year-old girls perceived a BMI below 18 (underweight) as ideal body size, possibly indicating that early adolescence may constitute a vulnerable developmental period for the onset of disordered eating-related cognitions and attitudes. A potential explanation may be that girls within this developmental phase typically start to experience changes in body composition (i.e., increase in body fat starting with puberty), after a period of typically having a relatively lean body during childhood, which may make this group particularly susceptible for a fear of body fat and the internalization of a pervasive thin-ideal. A general fear of growing or a fear of gaining secondary sex characteristics may also play a role during period and may explain the adoption of a pervasive thin-ideal among early-adolescent girls. With regard to symptoms of disturbed eating behavior, 15–17 year-old girls seemed to be most vulnerable. The results reflect age differences in the onset of different ED. The onset of anorexia (characterized by underweight or severe weight loss) typically lies in early adolescence and the onset of bulimia (characterized by disturbances in eating behavior such as binge eating and inappropriate compensatory behaviors) in late adolescence [63]. The results may reflect a developmental time course, in which cognitive-attitudinal distortions (e.g., adoption of pervasive female thin-ideal) in early-adolescence precede the onset and manifestation of symptoms of disturbed eating behavior during adolescence.

Several limitations should be acknowledged. First, the study has been conducted in a single state of Germany. Although North-Rhine Westphalia is Germany’s most populous state, the findings may not be entirely generalizable to the national population level and do not consider culture-related differences in body perceptions and body ideals. Moreover, body image perceptions and symptoms of disturbed eating behavior were self-reported by youth. It is possible that social desirability or response styles may have influenced the results. In addition, the cross-sectional design of the study does not allow to draw conclusions regarding temporal precedence or causality between study variables. While it is intuitive to assume that perceptual distortions precede the development of symptoms of disturbed eating behavior, it is also possible that children and adolescents with disturbed eating behavior develop perceptual distortions as a correlate of eating-related pathology. Moreover, it should be noted that the present study used single items to measure symptoms of ED, which may have limited psychometric properties. Also, the items did not assess the clinical severity of symptoms of disturbed eating behavior, as no clinical rating nor measures of frequency and severity were applied. In addition, it should be noted that the psychometric validity of the photographic figure rating has not been fully established. Yet, an expert-rating among mental health professionals indicated construct validity and previous studies have shown good test–retest validity and convergent validity of similar photographic figure rating scales [31–34]. Finally, it should be acknowledged that the present study did not control for a general underestimation bias. A body of research suggests that individuals tend to display under- instead of overestimation when asked to make judgements regarding size (e.g., when judging package or portion sizes, cf. Ordabayeva & Chandon [64]). Therefore, underestimation biases may constitute normative, hardwired cognitive errors, at least to a certain extent. The present study, however, shows that a strong bias towards underestimation of body size is associated with symptoms of disturbed eating behavior and psychological distress, indicating that strong perception biases are qualitatively different from common, benign errors. The present study also has several strengths including a large, heterogeneous sample of children and adolescents from all school types in Germany’s most populous state. In addition, the photographic rating, consisting of a variety of real women’s bodies, may have a better ecological validity in the assessment of body image perceptions than figure ratings used in previous studies. As the present rating used a larger number of female body photographs, the risk that a particular confounder was associated with a particular body size is decreased.

The present study suggests several recommendations for future research. First of all, prospective study designs are required to enable conclusions regarding temporal order to improve our understanding of the development and maintenance of ED. Future research may disentangle whether perceptual distortions constitute a risk factor predisposing youth towards the development of ED or merely a symptom of the ED. Furthermore, a better understanding of the frequency and the severity of symptoms of disturbed eating behavior among children and adolescents would be valuable. Future studies may investigate how often these symptoms are experienced by youth and whether they are associated with clinically significant distress or functional impairment. Finally, it would be interesting to investigate if perceptual distortions and symptoms of disturbed eating behavior can be modified by interventions. Possibly, psycho-education and cognitive interventions to modify normative misperceptions and perceptions of the thin-ideal may help to reduce eating-related pathology and prevent the development of ED among youth.

## Conclusions

In conclusion, the present study demonstrates that children and adolescents display a considerable perception bias (i.e., bias towards underestimation of female body size). Also, this study suggests the existence of a developmental time course, in which perceptual body-related distortions (e.g., body-related perception biases, internalization of pervasive thin-ideal) in early-adolescence may precede the onset and manifestation of symptoms of disturbed eating behavior during the course of adolescence. However, prospective studies will need to clarify temporal precedence between perceptions, cognitions, and behavior associated with eating-related pathology among youth in the future.

## Abbreviation

ED: eating disorders

## Footnotes

1 The present study solely assessed body image perceptions with regard to female bodies. For male bodies, no photograph material was available for reasons of feasibility. As the stimulus material pertained exclusively to female bodies, only female body image perceptions could be examined.

2 As a bias towards underestimation of the average female body size was present in nearly the entire sample and a clear cut-off for the definition of a perceptual bias is lacking, we used tertiles to compare groups scoring low compared to high on perception of average female body size.
